# Patient preferences do matter: a discrete choice experiment conducted with breast cancer patients in six European countries, with latent class analysis

**DOI:** 10.1017/S0266462323000168

**Published:** 2023-04-19

**Authors:** Eugena Stamuli, Sorcha Corry, Petter Foss

**Affiliations:** 1 Pharmecons Easy Access Ltd, York, UK; 2 Novartis Oncology Region Europe, Origgio, Italy

**Keywords:** Breast cancer, discrete choice experiment, trade-offs, latent class, Europe

## Abstract

**Objectives:**

The evolution of breast cancer (BC) treatments has resulted in tailored therapies for the different types and stages of BC. Each treatment has a profile of benefits and adverse effects which are taken into consideration when planning a treatment pathway. This study examines whether patients’ preferences are in line with what is considered important from decision makers viewpoint.

**Methods:**

An online discrete choice experiment was conducted in six European countries (France, Germany, Ireland, Poland, Spain, UK) with BC patients. Six attributes were included: overall survival (OS), hyperglycemia, rash, pain, functional well-being (FWB), and out-of-pocket payment (OOP). Sixteen choice sets with two hypothetical treatments and a “No treatment” option were presented. Data were analyzed with the use of heteroscedastic conditional, mixed logistic, and latent class models. Marginal rate of substitution (MRS) were estimated for OOP versus the rest of attributes to establish the ranking of preferences for each attribute.

**Results:**

Two hundred and forty-seven patients with advanced or metastatic BC and 314 with early-stage BC responded. Forty-nine percent of patients were < 44 years old and 65 percent had completed university education. The MRS of the analysis demonstrated that “severe pain” is the highest dis-preferred attribute level, followed by “severe impairment in FWB” and OS. Four classes of patients as “decision makers” were identified.

**Conclusions:**

This study suggests that there is heterogeneity in treatment preferences of BC patients depending on their sociodemographic and disease-related characteristics. In combination with clinical guidelines, patient preferences can support the selection and tailoring of treatment options.

## Introduction

Living longer and/or living better are the two main objectives of cancer treatments. The plethora of treatment modalities in oncology possess different profiles of survival benefit, disease progression, levels of toxicity, and impact on the health-related quality of life (HRQoL) of patients. Depending on the setting, that is, whether the new intervention has curative or noncurative intent, the clinical benefit is usually measured as prolongation of overall survival (OS), progression-free survival (PFS), event-free survival (EFS), or other surrogate end points. Similarly, HRQoL is measured via a variety of cancer-specific or generic quality of life (QoL) instruments. Importantly, for a cancer treatment to be considered of added clinical value, the magnitude of benefit for both survival and HRQoL is clearly outlined in relevant guidelines such as the European Society for Medical Oncology Magnitude of Clinical Benefit Scale (ESMO-MCBS) (1;2) and ASCO’s Value Framework (3). Hence, both attributes should be assessed on their own separate merits to establish whether a new cancer treatment should be adopted in clinical practice.

In the regulatory environment, traditionally a number of clinical end points such as OS, PFS, EFS, and disease-free survival (DFS) have been considered as adequate primary end points in confirmatory trials (4) with the HRQoL being considered only if good quality of data is ensured (5). There is, however a move toward transparently depicting the impact of both survival and the HRQoL in the benefit–risk framework and an urge to consider how relevant patient experience data can be incorporated into this framework to inform regulatory decision making (6). This stems from acknowledging the fact that patients might have different acceptability thresholds of risk and uncertainty in treatment outcomes, whether these be purely clinical or patient-related outcomes. Further, HRQL data are seen as complementary to a range of traditional indicators, which can provide information regarding both positive and negative patient experiences (5).

Overall, healthcare budget decision makers (“payers”) have a strong preference for OS data to measure the value of cancer medicines. The reimbursement decision making is based on a health technology assessment (HTA) process, which relies on a multitude of criteria, since aside from drug’s efficacy and safety it is also concerned with allocative efficiency. However, irrespective of the HTA paradigm, that is, whether the HTA is purely based on the cost–utility paradigm, the added clinical benefit and budget impact, or a multi-criteria decision analysis approach, most payers have a strong preference for OS data to measure the value of cancer medicines at the time of patient access decision making. In some HTA paradigms, OS is evaluated in combination with QoL (see quality-adjusted life-years) or as a sole, important end point. Nevertheless, evidence suggests that while QoL is deemed an important aspect in the clinical decision making, it is not well-reflected in HTA assessments (7;8), with OS being the driver of the decision making.

However, demonstrating OS benefits over a comparator drug might be hindered by a multitude of factors. Mature OS data (expressed as median OS) often are not available because of the length of the follow-up time that is required to obtain median OS data. In addition, numerous therapeutic options and an increasing number of treatment lines increase the complexity of isolating the effect of a particular therapy on OS due to the bias of confounding effects. Moreover, the availability of mature OS data differs by type and stage of cancer, that is, early, metastatic, or adjuvant setting. In rare cancers or personalized therapies, it may be difficult to obtain statistically significant OS data due to low sample size of patients. In the absence of mature OS data, PFS may be used as a surrogate end point where some quantification of the relationship between PFS and OS may be used to populate the economic model as an alternative to directly modeling OS from the trial data (9) although this approach is not adopted by all HTAs. In fact, there is a criticism of this approach, which leads to treatments being denied, restricted, or delayed due to immature OS data (10).

In order to avoid negative effects on the mortality and morbidity of patients due to the delay of new, promising treatments because of lack of mature OS data, a number of ways to mitigate payer uncertainty in HTA have been suggested (10;11) to assigning higher weight to end points such as improvement in the QoL and patient-related outcomes. In addition, incorporating patient preferences (PP) for treatment outcomes in the HTA decision making is a topic which is attracting a lot of attention for academic, industry, and policy-making environments (12;13). PP utilization can particularly assist in understanding what is more important to patients, since there might be the case that patients value HRQoL higher than OS (14). If this were the case, restrictions or delays of new treatments due to immature OS might not have a justified ground if the new treatments substantially improve patients’ HRQoL although cannot demonstrate OS due to aforementioned reasons.

Under this prism, a patient-preference study (15) was conducted in four European countries (France, Ireland, Spain, Poland) with the aim to assess what trade-off patients with breast cancer (BC) make for various treatment outcomes: PFS, levels of pain, impairment in functional well-being (FWB), and treatment toxicity (febrile neutropenia). The findings of this study suggest that BC patients value avoiding extreme pain or impairment in their FWB higher than extending the PFS. Patients are willing to trade-off treatment effectiveness (expressed in PFS) and out-of-pocket payments (OOPs) for perfect health states as described by the absence of pain and perfect FWB. The attributes in the study were considered independently; hence, any correlation between progression of disease and associated pain or reduced well-being is not taken into account. To capture the full importance of PFS as a patient relevant end point, it needs to be considered together with other related attributes as pain and well-being. This correlation might be different across cancer types and was out of scope for this study.

In this paper, a new study which builds on the previous one (15) is presented. The aim of the new study is to further explore PP when OS is included as an attribute instead of PFS, and whether preferences change depending on how the clinical outcome is measured. In addition, it explores whether PP differ based on their current health of state, as measured by EQ-5D instrument, as well as based on other sociodemographic characteristics. The inclusion of two additional countries (UK and Germany) broadens the perspective since the healthcare systems, patients’ contribution to their healthcare costs, and the HTA policies differ between countries.

## Methods

### Attribute selection

The methods for the attribute selection have been previously described in detail (15). Briefly, following good practices (16;17) an iterative approach involved: extensive literature review of guidelines on the BC treatments to create a list of candidate attributes, literature review of relevant clinical trials to capture the levels (magnitude of outcomes), and finalization of the attributes and levels via an advisory board. The advisory board comprised of clinicians, BC patients, and former health policy makers. The attributes and levels for the treatment options as well as the “opt-out of treatment” option are presented in Table 1.

**Table 1.** DCE2020 attributes and respective levels

Attribute	Levels for treatments	Opt-out of treatments	Reference (for assigning levels)
Overall survival (OS) in months	30, 35, 40, 45	20	(18–22)
Hyperglycemia (risk of occurring)	0%, 5%, 20%, 35%	0%	(23)
Rash (risk of occurring)	0%, 4%, 12%, 20%	0%	
Pain	Severe, moderate, no	Severe	Similar to Ref (15), based on descriptive levels of EQ5D-3 L
Functional well-being (FWB)	Severe, Moderate, No	Severe	Similar to Ref (15) based on descriptive levels of EQ5D-3 L
Out-of-pocket payment (OOP) in Euros plus respective countries	€0, €3000, €5000, €8000 Based on purchasing power parity (PPP)^a^ Spain (€):0, 2807, 4678, 7485 France (€):0, 3277, 5462, 8740 Ireland (€):0, 3567, 5944, 9511 Poland (PNL): 0, 7838, 13063, 20900 Germany (€):0, 3302, 5503, 8805 UK (£): 0, 3047, 5079, 8126	0	Similar to Ref (15)

a The OOP was adjusted for each country based on the PPP.

Both hyperglycemia and rash are adverse events that have been observed in the Alpelisib trial (24), so it was deemed important to explore the preferences of the BC patients for treatments that exhibit these characteristics. OS attribute replaced the PFS of the previous study (15). The rest of the attributes remained unchanged.

### Experimental design

The experimental design for DCE2020 was developed in SAS (version 9.4, SAS Institute Inc., Cary, NC, USA), based on D-efficiency criterion, for estimating main effects only and following the “dual response” approach. The respondents were asked to choose from a set of available alternatives which included the options: treatment A, treatment B, and no treatment/opt-out. Supplementary Figure S1 presents an example of how the discrete choice experiment (DCE) questions were presented in the survey. The questionnaire included sixteen choice sets. Two additional choice sets, one duplicate and one choice set including a dominant scenario, were included in the questionnaire to test for consistency and rationality in choices of respondents.

In addition to the DCE questionnaire, sociodemographic and disease-related questions were included: age, menopausal status, level of education, income group and working status, stage of cancer, and type of treatment that the respondent is receiving or has received in the past. In addition, the EQ-5D questionnaire was administered, as developed by EuroQoL (31) in the native language of each participating country, to capture the current health state of the respondents. The questionnaire was developed in English and was translated into French, German, Spanish, and Polish by professional medical translators, by applying forward-back translation process to the source language of the questionnaire (i.e., English). Cognitive debrief was performed with two native speakers for each language to ensure the validity and the cultural relevance of the translation.

### Sample size and patient recruitment

Patients ≥18 years old, with BC, and from the following countries: France, Ireland, Poland, Spain, Germany, and UK were recruited. Quota sampling was followed to achieve a representation of the advanced/metastatic BC as ≥50 percent of the sample. A sample size *N* of sixty-three respondents per participating country was calculated based on the following formula (25):
[1]

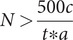

where *c* is the number of analysis cells, or the largest number of levels for any attributes, for a design which includes only main effects, *t* is the number of choice tasks, and *a* is the number of alternatives.

Other rules for calculating the sample size, for example, the rule of thumb proposed by Pearmain et al. (26), suggest that, for DCE designs, sample sizes over 100 are able to provide a basis for modeling preference data, whereas Lancsar and Louviere (27) mention that “our empirical experience is that one rarely requires more than 20 respondents per questionnaire version to estimate reliable models, but undertaking significant post hoc analysis to identify and estimate covariate effects invariably requires larger sample size.” Based on the above, the aim was to recruit a sample of 100 patients per country.

The survey was designed as an internet-based, self-completion survey. The sample was recruited via a market research company, Dynata (www.dynata.com). For Ireland, Marie Keating Foundation (https://www.mariekeating.ie/) was instrumental in assisting Dynata with identification of the right population in Ireland, due to extremely small number of BC patients. Ethics approval was provided by HML Institutional Review Board (https://www.healthmedialabirb.com/), an independent research review board, acting at international level.

The questionnaire was piloted with 125 members of general population, or approximately 20 respondents per country. We anticipated that patient recruitment would be affected by COVID-19 pandemic; hence, general population was used instead of patients to ensure that valuable and difficult to recruit patient sample was not used for piloting purposes, rather for the main survey. The survey was adjusted for the pilot sample, by removing all disease-specific questions (e.g., stage and hormonal type of cancer, present, and prior treatments). The pilot DCE data were fully analyzed prior to proceeding with patient recruitment, to establish whether the findings are intuitive and expected, and the questionnaire is appropriately designed.

### Data analysis

For the sociodemographic and disease-specific characteristics, descriptive statistics are presented for the total sample as well as for each country separately. Utility values were derived from the EQ-5D data by applying the countries specific tariffs via *eq5d* command in STATA (28). French and Polish tariffs were taken from Chevalie et al. (29) and Golicki et al. (30), respectively, since these are not available in the *eq5d* command. The UK tariff was applied to data of the Irish respondents. EQ-5D data were additionally analyzed as “Health Profiles” following the guidelines provided by EuroQoL (31) presenting the number and percentage of patients reporting each level of problem on each dimension of the EQ-5D. This analysis provides a more in-depth information on the aspects of patients’ health that have been most affected by their condition, or improved by treatment (31).

For the analysis of the choice data, several econometric models were utilized to explore preferences and how the treatment attributes as well as sociodemographic data impact patients’ choices. The first analysis was conducted with the use of the conditional logit model (clogit), since this is by far the most widely used DCE model, due to its simplicity and ease of application (32). Starting with clogit provides a very good picture on the quality of the data set and, further, allows for variations and extensions of the basic specification for more advanced analysis. Data for all six countries were pooled; hence, a parameterized heteroscedastic conditional logit (HCL) model was fitted (33;34) to account for scale heterogeneity between the countries.

There are three underlying assumptions of the conditional logit model, that is, independence of irrelevant alternatives, the independent and identically distributed across observations error term, and the lack of unobserved preference heterogeneity across respondents (32). Given the drawbacks of this model, a mixed logit model was additionally fitted to account for taste heterogeneity (35). The mixed logit model extends the standard conditional logit model by allowing one or more of the parameters in the model to be randomly distributed and the coefficients in the model to vary across decision makers. Hence, individual-specific variations in preferences can be captured. The model was fitted via mixlogit command in STATA 16 (36), by assuming a normal distribution for all the covariates bar OOP, which was specified to have fixed coefficient. Lastly, the effect of sociodemographics and health status (i.e., age, education, stage of cancer, and EQ-5D-based utility score) on the PP was investigated by including interaction terms of attributes with the respective variables.

A third analysis involved the use of latent class model, which assumes that there is a distinct number of classes based on the different types of preferences; the preferences are homogeneous within each class, although there is heterogeneity of preferences across the classes specified in the model. Latent class identifies subgroups of choices, and membership of those subgroups can then be assessed based on sociodemographic and clinical factors. This identifies sources of heterogeneity, rather than subgroups per se, that is, the classes group respondents based on their nonobservable characteristics such as willingness to avoid high levels of pain or OOP, or conversely, willingness to experience higher OS. Since the classes are unknown a priori, the optimal number of classes utilized in the latent class analysis (LCA) is identified by examining goodness-of-fit of the data through inspection of the Bayesian information criterion (BIC) and consistent Akaike information criterion (AIC). The most appropriate number of classes is the one which minimizes these two criteria (37). This analysis was implemented with the use of lclogit2 and lclogitml2 (38). In addition, to estimate how well the model performs in differentiating several classes of preferences, the highest posterior probability of class membership was computed (as the average over respondents).

The trade-offs that responders are willing to make between attributes are defined as the marginal rate of substitution (MRS). The MRS was estimated by using the β-coefficients which are derived from conditional and mixed logit models, without interaction effects. The MRS quantifies how much the individuals are willing to pay as OOP money for improvements in the other attributes. Similarly, by using the β-coefficient for the OS attribute, the MRS estimates how much of OS one is willing to forgo for improvements in other attributes, for example, less pain, better FWB, smaller chance of treatment-related hyperglycemia, or rash occurring. The MRS analysis guides the rank ordering of the various attributes. In addition, it provides a way of comparing the choices from the participating countries, overcoming potential issues with preference heterogeneity.

Stata/MP 16.0 for Windows statistical package (StataCorp. 2019. College Station, TX: StataCorp LP) was used for all the analyses.

## Results

### Sociodemographic and disease-specific characteristics

A total of 561 BC patients were recruited (France 119, Germany 106, Ireland 57, Poland 79, Spain 100, UK 100). Key sociodemographic and disease-related characteristics are presented in Supplementary Table S1. Twenty eight percent of the sample were between 35 and 44 years old, 65 percent had completed university education, and 56 percent were working at the time of the survey. Following the American Joint Committee on Cancer TNM staging system (39) and recommended by ESMO (40), 247 respondents (44 percent of the sample) had advanced or metastatic BC (AMBC), while 314 (56 percent) were in early stages (adjuvant or localized). Of the 247 patients with AMBC, 103 (42 percent) had only one site of metastasis, while the other patients (N = 144, 58 percent) had two or more sites.

With regard to the EQ-5D data, 61, 69, and 47 percent of the sample reported no problems in the dimensions “mobility,” “self-care,” and “usual activities,” respectively. Sixty-six percent and 51 percent of the respondents reported having moderate pain and anxiety, respectively (Supplementary Table S2). Pain, one of five domains of the EQ-5D, has the largest proportion of patients (78 percent of the total sample) in an impaired state (either in “moderate” or “extreme” pain) compared to the other four domains.

### Results of choice data analysis

#### HCL and mixed logit models

The results of the HCL and mixed logit models are shown in Table 2. The significant scale coefficient for Ireland in the HCL model indicates that there is scale heterogeneity versus the reference country Poland. For the rest of the countries, the scale coefficients are nonsignificant.

**Table 2.** Results of heteroscedastic conditional and mixed logit models

	HCL				Mixed logit			
	b	SE	95%CI		b	SE	95%CI	
Alternative specific constant ASC = 1 for treatment, 0 for opt-out	.202*	.080	.045	.360	1.016***	.118	.785	1.248
OOP (‘000 Euros)	−.017*	.007	−.031	−.004	−.030***	.006	−.041	−.020
OS	.015***	.004	.008	.022	.019***	.004	.011	.026
Hyperglycemia	−.007***	.001	−.010	−.004	−.011***	.001	−.014	−.008
Rash	−.005***	.001	−.008	−.002	−.007**	.002	−.011	−.003
No pain reference								
Severe pain	−.543***	.078	−.695	−.391	−.807***	.059	−.922	−.692
Moderate pain	.037	.027	−.017	.090	.068	.040	−.011	.147
No impairment FWB reference								
Severe impairment FWB	−.562***	.075	−.709	−.414	−.758***	.055	−.865	−.651
Moderate impairment FWB	−.090***	.026	−.141	−.038	−.118**	.039	−.194	−.042
Het (Poland as reference)								
COUNTRY:Germany	.203	.159	−.109	.514				
COUNTRY:UK	.021	.174	−.321	.363				
COUNTRY:Spain	.012	.185	−.351	.374				
COUNTRY:Ireland	.691***	.167	.363	1.019				
COUNTRY:France	−.133	.200	−.525	.259				
SD								
ASC					1.710***	.109	1.496	1.924
OS					.058***	.004	.049	.067
Hyperglycemia					.017***	.002	.013	.021
Rash					−.003	.004	−.011	.005
Severe pain					1.072***	.057	.959	1.184
Moderate pain					.068	.100	−.128	.265
Severe impairment FWB					.892***	.056	.783	1.002
Moderate impairment FWB					.053	.058	−.061	.168
Number of observations	26,928			26,928				
Chi-squared	137.473			2,408.444				
Model degrees of freedom	14			17				
Log-likelihood	−8,421.088			−7,285.603				
AIC	16,870.18			14,605.21				
BIC	16,984.99			14,744.62				

* *p* < .05;

** *p* < .01;

*** *p* < .001.

In both models, the regression coefficients related to all the attributes, bar moderate pain, are significant (at either 0.001 or 0.01 level), indicating that all the attributes are important in the choice of treatment. The direction of the effects is also as expected: higher OOP or risk of adverse events (hyperglycemia and rash) decrease the probability of a treatment being chosen (negative coefficients). Higher levels of these three attributes are associated with disutility to the patients. Positive coefficient of the alternative specific constant indicates that patients have preference for receiving a treatment.

The larger negative coefficient of hyperglycemia compared to rash indicates that participants have larger dis-preference for hyperglycemia than rash (−0.011 vs −0.007 from mixed logit model). For pain and FWB attributes, worse levels than “no pain” and “no impairment in FWB” are associated with disutility as denoted by negative coefficients. Nonsignificant coefficient for “moderate pain” denotes that patients are indifferent to this level compared to “no pain” level. Each additional month of OS is associated with an increase in preference weight, that is, the utility associated with each level or unit of the attribute of 0.015 (HCL model) and 0.019 (mixed logit model). The significant standard deviations in the mixed logit model indicate that there is considerable heterogeneity across the individuals (taste heterogeneity) for the all the attributes bar the “moderate” levels of pain and impairment in FWB, and rash.

Finally, based on the MRS (Table 3) it is important to note that although the ranking of the attributes’ importance is the same between the two models, the magnitude of the MRS differs. The values of MRS are smaller in the mixed logit results, potentially due to accounting for preference heterogeneity in this analysis. The negative values of MRS indicate that patients are willing to pay OOP to avoid adverse events (rash, hyperglycemia) or avoid moderate and severe levels of pain and impairment in FWB. Based on the results of mixed logit model, patients are willing to pay the largest amount of OOP for avoiding severe level of pain (around €26 thousand for a pain-free year) followed by FWB (around €25 thousand for 1 year in perfect FWB). The effect of sociodemographics and health status (i.e., age, education, stage of cancer, and EQ-5D-derived utility score) on the PP, which was investigated by including interaction terms of attributes with the respective variables, is presented in Supplementary Table S4. Interestingly, this analysis shows that patients in better health states (higher EQ-5D-derived utility scores) have higher dis-preference for severe levels of pain or FWB. This implies either some level of adaptation effect of patients who are in worse health states or indeed, the pain can be viewed at quite differently if one feels it in contrast to imagining it.

**Table 3.** Marginal rate of substitution

	Heteroscedastic model			Mixed logit		
	Mean (in thousand Euros)	95% CI		Mean (in thousand Euros)	95% CI	
OS	.88	.22	1.54	.62	.31	.94
Hyperglycemia	−.42	−.71	−.13	−.38	−.54	−.21
Rash	−.30	−.55	−.05	−.23	−.38	−.07
Severe pain	−31.38	−52.55	−10.20	−26.61	−36.59	−16.63
Moderate pain	2.12	−1.33	5.57	2.25	−.62	5.12
Severe impairment FWB	−32.49	−54.78	−10.20	−25.01	−34.44	−15.58
Moderate impairment FWB	−5.18	−9.65	−.71	−3.90	−6.69	−1.10

#### Latent class analysis

The LCA was conducted with four classes. Although the AIC and BIC values were better for higher number of classes (Supplementary Table S3), there were convergence problems; hence, the analysis was conducted with four classes (Table 4). The mean highest posterior probability is 92.4 percent, which demonstrates that the model performs well in distinguishing among different underlying taste patterns for the observed choice behavior.

**Table 4.** Latent class analysis results

	Class 1 (Pr = 36.19%) *N* = 203 Rational decision makers			Class 2 (Pr = 9.45%) *N* = 53 Treatment avoiders			Class 3 (Pr = 38.50%) *N* = 216 Money indifferent			Class 4 (Pr = 15.86%) *N* = 89 Pay anything for longer life		
	France 18% Germany 22% Ireland 4% Poland 18% Spain 18% UK 20%			France 43% Germany 13% Ireland 2% Poland 13% Spain 19% UK 9%			France 22% Germany 16% Ireland 16% Poland 10% Spain 20% UK 15%			France 12% Germany 21% Ireland 15% Poland 16% Spain 11% UK 25%		
	b	SE	95 % Ci	b	SE	95 % Ci	b	SE	95 % CI	b	SE	95 % CI
ASC (1 for treatment, 0 for opt-out)	.651***	.126	.405, .898	−1.154***	.302	−1.746, −0.561	1.484***	.248	.998, 1.971	1.373***	.334	.718, 2.028
OOP (‘000 Euros)	−.073***	.01	−.092, −.053	−.183***	.041	−.264, −.103	−.013	.013	−.038, −.012	.100***	.018	.064, .136
OS	.010*	.005	.001, .019	.002	.013	−.023, .027	.016*	.006	.004, 0.29	.075***	.01	.055, .094
Hyperglycemia	−.008***	.002	−.012, −.004	−.023***	.006	−.034, −.011	−.019***	.003	−.024, −0.014	.002	.004	−.006, .010
Rash	−.008*	.003	−.015, −.002	.002	.01	−.017, .021	−.019***	.004	−.028, −.011	.002	.005	−.007, .012
No pain -reference level												
Severe pain	−.137*	.062	−.259, −.016	−.763***	.202	−1.159, −.366	−1.771***	.124	−2.014, −1.529	−.081	.102	−.281, .119
Moderate pain	.099	.066	−.030, .229	−.05	.204	−.450, .351	−.043	.077	−.195, .109	.059	.108	−.152, .270
No impairment FWB reference												
Severe impairment FWB	−.321***	.069	−.457, −.185	.055	.182	−.301, .411	−1.829***	.122	−2.068, −1.590	−.023	.112	−.243, .197
Moderate impairment FWB	−.05	.06	−.168, .069	.086	.203	−.312, .483	−.533***	.098	−.725, −.342	.041	.095	−.146, .227
Number of observations	26,928											
Model degrees of freedom	39											
Log-likelihood	−7,219.89											
AIC	14,517.79											
BIC	14,837.62											

* *p* < .05;

*** *p* < .001.

AIC, Akaike information criterion; BIC, Bayesian information criterion; FWB, functional well-being; OOP, out-of-pocket payment; OS, overall survival.

The LCA resulted in two large groups of patients: class 1 and class 3, with 203 and 216 patients, respectively. Class 2 and class 4 were smaller (with 53 and 89 patients, respectively). Additionally, the first row of Table 4 presents the proportion of patients from each country in each class. For example, 22 percent of patients comprising class 1 come from Germany, 43 percent of class 2 come from France, 22 percent of class 3 come from France, and 25 percent of class 4 come from the UK, showing the highest percentage coming from each country.

A brief description of preferences for each class is provided:

- Class 1 (N = 203): Patients in this class prefer to receive cancer treatment, have dis-preference for OOP, treatment-related adverse events (hyperglycemia and rash), and severe levels of pain and impairment in FWB. They are indifferent to moderate levels of pain and impairment of FWB, as denoted by the nonsignificant coefficients for these two levels.
- Class 2 (N = 53): Patients in this class are “treatment avoiders” as the ASC has a negative value and is statistically significant. In other words, patients have negative utility when they receive treatment. The only important attributes for these patients are OOP, hyperglycemia, and severe pain.
- Class 3 (N = 216): The preferences of patients in this class are similar to those allocated in class 1. Severe pain and severe impairment FWB are important attributes for class 3. The only difference relates to this class being indifferent to OOP as indicated by the nonsignificant coefficient for this attribute. The amount of money that these patients should pay as OOP for their treatment is not a decision driver for the treatment choice.
- Class 4(N = 89): This group of patients makes counterintuitive choices, as they receive higher utility from paying OOP for their treatment. This is probably an indication that this group of patient associates higher payment with better treatment outcomes (41). Interesting to note that 25 percent of this class comes from the UK where the health care is fully funded by the national health services and the option of private health care is limited.

**Figure 1.**
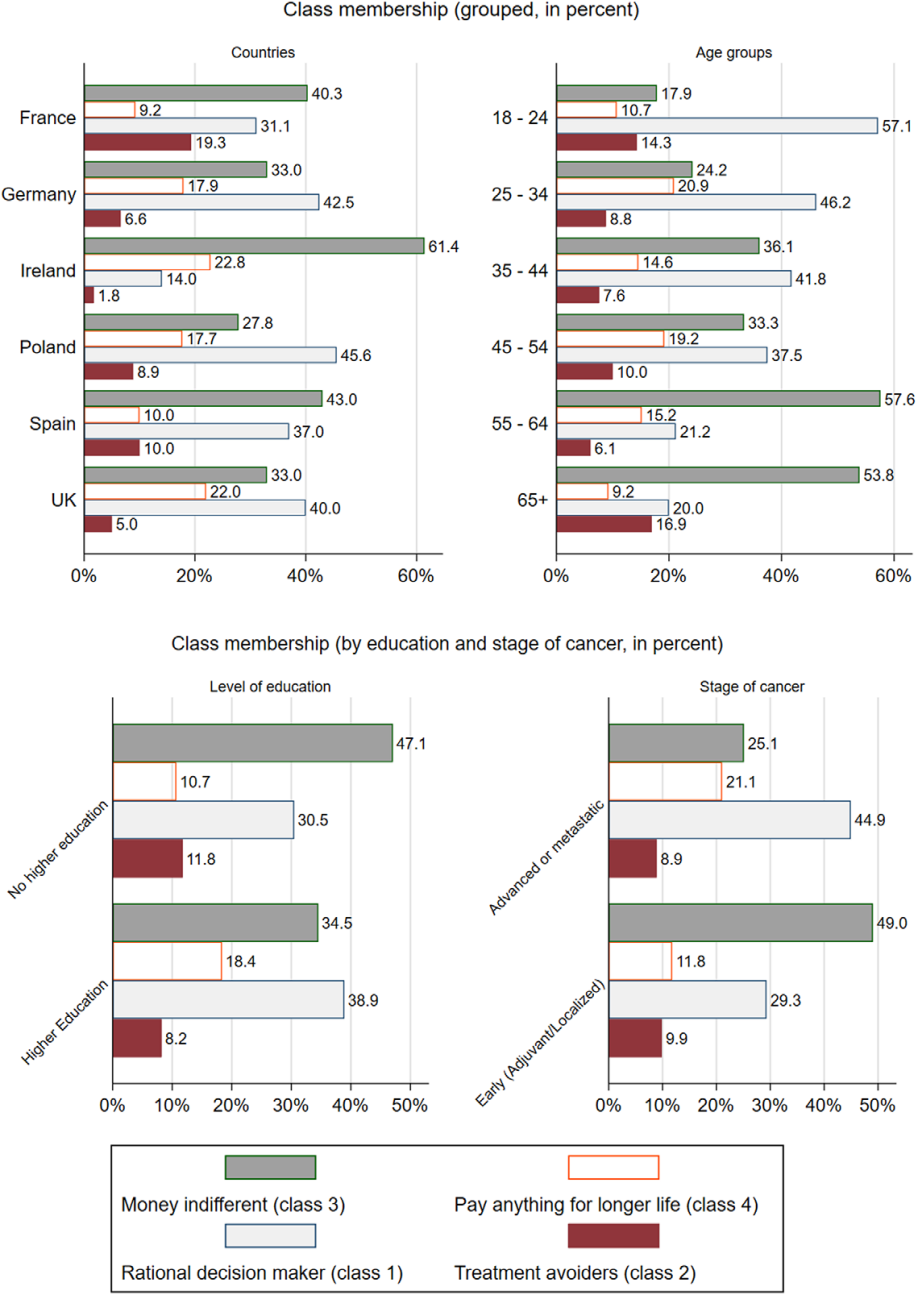
Class membership per patient characteristics.

Figure 1 presents an overview of how the total sample is split across the four classes by country of origin, age group, stage of cancer, and level of education. For example, the French sample has the following split: 40 percent belong to class 3, 9 percent to class 4, 31 percent to class 1, and 19 percent to class 2. Additionally, most patients with higher education (38.9 percent) and AMBC (44.9 percent) fall in the class 1 (rational decision makers). Supplementary Table S5 presents the mean utilities per latent class, derived from applying country-specific EQ-5D tariffs.

## Discussion

This study reports on the preferences of BC patients on treatment outcomes. It is an extension of a previous study (16) conducted in four European countries – France, Spain, Ireland, and Poland – which concluded that patients are willing to forgo months of PFS if they were to achieve states of no pain and perfect FWB. The current study further explored what the preferences are when the clinical outcome is defined as “hard outcome,” that is, OS, in addition to the existing attributes from previous study (16): pain, FWB, toxicity (expressed as rash and hyperglycemia), and OOP. UK and Germany were added in the second wave of the study, along with the countries included in the first wave. Expanding the pool of countries to include the UK and Germany gave a broader representation of different healthcare systems, including varying methods of financing (purely through taxation, private insurance, or a combination of both), the format used for HTA reimbursement decisions, and whether patients accessing health care are required to contribute to the healthcare costs or have free health care at the point of access.

Topics on the design of the DCE and the analysis of data, as well as the limitations of the study, have been thoroughly laid out and explored in the wave one paper (16). The new analytical element in the current paper relates to the additional use of latent class model, which provides more granularity and insights on the patients’ preferences for the various treatment outcomes. In the first instance, the analysis based on the mixed logit model demonstrated that, overall, patients value higher avoiding severe levels of pain, followed by severely impaired FWB and OS. It also captured the preference heterogeneity for the all the attributes bar the “moderate” levels of pain and impairment in FWB, and rash.

The MRS demonstrated that patients are willing to pay the largest amount of OOP for avoiding severe level of pain (around €26 thousand for a pain-free year) followed by FWB (around €25 thousand for 1 year in perfect FWB). This finding is at odds with the priorities which HTA set through their criteria used for reimbursement decisions. In other words, what is perceived as an important outcome and a decision driver from the HTA perspective (i.e., OS), might be of secondary importance to patients, if they are concerned about the level of pain and FWB. The study adds to the existing body of evidence on PP which shows that, in many instances in oncology, clinical outcomes such as OS and PFS are of secondary importance to patients (42;43) if the HRQoL is extremely compromised.

Furthermore, the results of the LCA show a more complex picture, which might be very important to health policy makers. The results of the LCA reveal a classification of patients into four distinct groups: those who choose treatments as expected (class 1; 36 percent); those who avoid treatments (class 2; 9.5 percent), possibly due to a preconception it may not benefit or even deteriorate their overall well-being, also known as “refuseniks” (44). Interestingly, avoiding severe pain is the most highly valued outcome for the “treatment avoiders”; hence, these patients might associate any treatment with induction of pain. Another group of patients are indifferent to cost (class 3, 38.5 percent); therefore, the OOP is not a decision driver and they prioritize avoiding severe levels of FWB impairment, followed by pain. Finally, a subset of patients (class 4, 15.9 percent) perceive OOP expenses as an indicator of superior treatment outcomes, and therefore choose options with higher costs. Notably, this group considers OS to be the most critical outcome. Additionally, the distribution of patients across different classes varies between countries, suggesting that the decision-making behavior of patients can be influenced by factors such as models of healthcare financing and drug reimbursement processes.

The findings from the LCA are crucial in highlighting the diversity of patients’ preferences and the fact that there is no “average patient.” This raises questions on whether regulatory or reimbursement decisions for new cancer treatments, which are mostly taken on aggregate, population level can equally benefit separate patients’ groups. In practice, reimbursement decisions are often based primarily on the existence of mature OS data (10), which can overlook the preferences of specific patient groups who might benefit from the treatment in alternative, patient relevant end points. Even more so, when these end points are congruent with what patients consider to be the most significant outcome from their own viewpoint. The lack of OS data, due to many reasons, as outlined in the introduction of this paper, and therefore the rejection of new treatments, results in penalizing specific group of patients who have different expectations (14) from their treatments. This underscores the necessity for innovative reimbursement decision-making approaches that prioritize a patient-centered perspective, by considering the diverse needs and preferences of different patient groups. One possible way to implement this is through decisions that result in differential access to the technology, reflecting different “classes” of patients.

Indeed, the integration of PP into decision making has gained increasing attention at various jurisdictions, including HTA decision making (13), regulatory (6), and research (45) which is exploring methods of incorporating patient perspective and preferences throughout the life cycle of medicines. The consensus is that PP are seen as drivers of patient’s adherence to treatment, satisfaction with it, and experienced outcomes (46). In addition, they provide a mechanism which could allow broader elements of value (see “value flower” of ISPOR) (47) to be more explicitly incorporated in the evaluation of the new technologies. Importantly, respecting and incorporating PP in treatment decisions constitute one of the eight principles for a true patient-centric care (48), which is the goal of many healthcare systems and decision makers nowadays.

Incorporating PP into decision making is an ongoing area of research, and there is currently no universally accepted gold standard for doing so. This is due in part to the diverse nature of PP and the complexity of decision-making processes. However, general steps are provided and can be adapted to suit specific contexts and situations (49): identification of the need for PP in the various decision-making spaces (regulatory, HTA bodies), involvement of decision makers, and other stakeholders in the design, conduct, and dissemination of patient-preference studies. This helps ensure that decision makers’ needs and concerns are addressed, and that results are presented in a useful way. Other stakeholders such as patient advocates, clinicians, and industry representatives can also provide important perspectives. Overall, engaging with decision makers and stakeholders is important to ensure that patient-preference studies are relevant, useful, and impactful in real-world decision making.

## Supplementary material

For supplementary material accompanying this paper visit https://doi.org/10.1017/S0266462323000168. click here to view supplementary material
