# Supplementary material for: Patient preferences do matter: a discrete choice experiment conducted with breast cancer patients in six European countries, with latent class analysis
Source: Int J Technol Assess Health Care. 2023 Apr 19;39(1):e21. doi: 10.1017/S0266462323000168 (PMC11574541; doi:10.1017/S0266462323000168)
Supplement: Supplementary file 1 [file S0266462323000168sup001.docx]

# Appendix

Figure 1: DCE example question


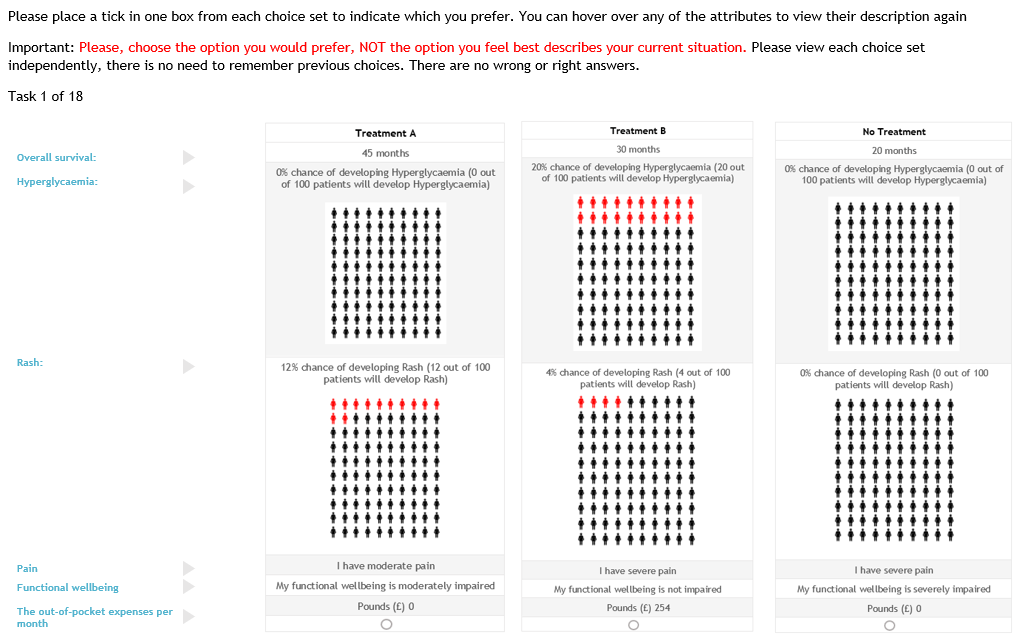


Table 1: Sociodemographic and disease specific characteristics of respondents

|  | **COUNTRY N (%)** | | | | | | |
| --- | --- | --- | --- | --- | --- | --- | --- |
|  | France | Germany | Ireland | Poland | Spain | UK | **Total** |
|  | N=119(%) | N=106(%) | N=57(%) | N=79(%) | N=100(%) | N=100(%) | N=**561(%)** |
| **Utilities captured by EQ-5D** | | | | | | | |
| (Mean, std.dev) | 0.55 (0.38) | 0.73 (0.27) | 0.68 (0.27) | 0.78 (0.22) | 0.65 (0.29) | 0.47 (0.39) |  |
| **Age group** | | | | | | | |
| **18 - 24** | 0(0) | 2(2) | 1(2) | 12(15) | 4(4) | 9(9) | **28(5)** |
| **25 - 34** | 5(4) | 20(19) | 3(5) | 19(24) | 19(19) | 25(25) | **91(16)** |
| **35 - 44** | 30(25) | **37(35)** | 6(11) | **25(32)** | **27(27)** | **33(33)** | **158(28)** |
| **45 - 54** | **32(27)** | 14(13) | 18(32) | 11(14) | **27(27)** | 18(18) | **120(21)** |
| **55 - 64** | 26(22) | 20(19) | **22(39)** | 8(10) | 19(19) | 4(4) | **99(18)** |
| **65+** | 26(22) | 13(12) | 7(12) | 4(5) | 4(4) | 11(11) | **65(12)** |
| **University education** | | | | | | | |
| **No** | 59(50) | 46(43) | 13(23) | 16(20) | 22(22) | 31(31) | **187(33)** |
| **Yes** | **59(50)** | **60(57)** | **41(72)** | **60(76)** | **76(76)** | **69(69)** | **365(65)** |
| **Prefer not to answer** | 1(1) | 0(0) | 3(5) | 3(4) | 2(2) | 0(0) | **9(2)** |
| **Employment status** | | | | | | | |
| **Housewife** | 6(5) | 3(3) | 5(9) | 2(3) | 9(9) | 2(2) | **27(5)** |
| **Not working due to long term sickness** | 12(10) | 21(20) | 8(14) | 8(10) | 22(22) | 14(14) | **85(15)** |
| **Retired** | 36(30) | 13(12) | 9(16) | 5(6) | 7(7) | 10(10) | **80(14)** |
| **Unemployed** | 16(13) | 6(6) | 2(4) | 3(4) | 7(7) | 8(8) | **42(7)** |
| **Working** | **48(40)** | **61(58)** | **30(53)** | **59(75)** | **50(50)** | **65(65)** | **313(56)** |
| **Other** | 1(1) | 2(2) | 3(5) | 2(3) | 5(5) | 1(1) | **14(2)** |
| **Stage of cancer** | | | | | | | |
| **Advanced/metastatic (Stage 3-4)** | 54(45) | 50(47) | 26(46) | 17(22) | 50(50) | 50(50) | **247(44)** |
| **One Site of metastasis** | 16(30) | 18(36) | 10(38) | 12(71) | 34(68) | 13(26) | **103(42)** |
| **Two or more sites of metastasis** | 38(70) | 32(64) | 16(62) | 5(29) | 16(32) | 37(74) | **144(58)** |
| **Early (Adjuvant/ Localized) (Stage 1 - 2)** | 65(55) | 56(53) | 31(54) | 62(78) | 50(50) | 50(50) | **314(56)** |

Table 2 : ΕQ-5D-3L dimensions

|  | France | | | Germany | | Ireland | | Poland | Spain | | UK | | **Total** | |
| --- | --- | --- | --- | --- | --- | --- | --- | --- | --- | --- | --- | --- | --- | --- |
| **Domain** | N=119(%) | | | N=106(%) | | N=57(%) | | N=79(%) | N=100(%) | | N=100(%) | | N=**561(%)** | |
| **Mobility** | | | | | | | | | | | | | | |
| **I have no problems in walking about** | | 73(61) | 63(59) | | 41(72) | | 61(77) | | | 56(56) | | 50(50) | | **344(61)** |
| **I have some problems in walking about** | | 38(32) | 41(39) | | 16(28) | | 17(22) | | | 42(42) | | 43(43) | | 197(35) |
| **I am confined to bed** | | 8(7) | 2(2) | | 0(0) | | 1(1) | | | 2(2) | | 7(7) | | 20(4) |
| **Self-care** | | | | | | | | | | | | | | |
| **I have no problems with self-care** | | 86(72) | 71(67) | | 51(89) | | 64(81) | | | 67(67) | | 49(49) | | **388(69)** |
| **I have some problems washing or dressing myself** | | 28(24) | 33(31) | | 5(9) | | 14(18) | | | 32(32) | | 44(44) | | 156(28) |
| **I am unable to wash or dress myself** | | 5(4) | 2(2) | | 1(2) | | 1(1) | | | 1(1) | | 7(7) | | 17(3) |
| **Usual activities** | | | | | | | | | | | | | | |
| **I have no problems with performing my usual activities** | | 58(49) | 47(44) | | 26(46) | | 52(66) | | | 43(43) | | 37(37) | | **263(47)** |
| **I have some problems with performing my usual activities** | | 49(41) | 56(53) | | 28(49) | | 25(32) | | | 50(50) | | 56(56) | | 264(47) |
| **I am unable to perform my usual activities** | | 12(10) | 3(3) | | 3(5) | | 2(3) | | | 7(7) | | 7(7) | | 34(6) |
| **Pain/discomfort** | | | | | | | | | | | | | | |
| **I have no pain or discomfort** | | 30(25) | 18(17) | | 20(35) | | 20(25) | | | 16(16) | | 23(23) | | 127(23) |
| **I have moderate pain or discomfort** | | 75(63) | 75(71) | | 34(60) | | 50(63) | | | 75(75) | | 59(59) | | **368(66)** |
| **I have extreme pain or discomfort** | | 14(12) | 13(12) | | 3(5) | | 9(11) | | | 9(9) | | 18(18) | | 66(12) |
| **Anxiety/depression** | | | | | | | | | | | | | | |
| **I am not anxious or depressed** | | 58(49) | 32(30) | | 22(39) | | 27(34) | | | 35(35) | | 30(30) | | 204(36) |
| **I am moderately anxious or depressed** | | 41(34) | 63(59) | | 30(53) | | 40(51) | | | 59(59) | | 52(52) | | **285(51)** |
| **I am extremely anxious or depressed** | | 20(17) | 11(10) | | 5(9) | | 12(15) | | | 6(6) | | 18(18) | | 72(13) |

Table 3: Information criteria for various number of classes including constant

| Classes | LLF | Nparam | AIC | BIC |
| --- | --- | --- | --- | --- |
|  |  |  |  |  |
| 2 | -7623.87 | 19 | 15387 | 15368 |
| 3 | -7354.68 | 29 | 14921.92 | 14892.92 |
| 4 | -7211.14 | 39 | 14708.14 | 14669.14 |
| 5 | -7121.92 | 49 | 14603 | 14554 |
| 6 | -7004.56 | 59 | 14441.57 | 14382.57 |

Table 4: Mixed logit model with interaction terms for patient characteristics

|  | Age | | | | Education | | | Stage | | | Utility score | | |
| --- | --- | --- | --- | --- | --- | --- | --- | --- | --- | --- | --- | --- | --- |
| Variable | b | se | ci95 | | b | se | ci95 | b | se | ci95 | b | se | ci95 |
| OOP_new | -0.008 | 0.007 | -0.023,0.006 | | -0.067*** | 0.01 | -0.087,-0.047 | -0.060*** | 0.008 | -0.075,-0.044 | 0.003 | 0.012 | -0.021,0.027 |
| const | 0.890*** | 0.123 | 0.65,1.13 | | 0.833*** | 0.11 | 0.617,1.049 | 0.933*** | 0.122 | 0.693,1.172 | 0.845*** | 0.112 | 0.625,1.064 |
| OS | 0.011** | 0.004 | 0.003,0.018 | | 0.023*** | 0.006 | 0.01,0.036 | 0.023*** | 0.005 | 0.012,0.033 | 0.002 | 0.006 | -0.01,0.015 |
| Hyperglycaemia | -0.007*** | 0.002 | -0.011,-0.003 | | -0.017*** | 0.003 | -0.022,-0.012 | -0.018*** | 0.002 | -0.022,-0.014 | 0.001 | 0.003 | -0.005,0.007 |
| Rash | -0.006 | 0.003 | -0.011,0 | | -0.011** | 0.004 | -0.019,-0.004 | -0.005 | 0.003 | -0.01,0.001 | -0.009 | 0.004 | -0.017,0 |
| Pain_severe | -0.430*** | 0.07 | -0.568,-0.293 | | -1.060*** | 0.102 | -1.261,-0.859 | -1.099*** | 0.079 | -1.253,-0.944 | -0.192 | 0.116 | -0.419,0.034 |
| Pain_moderate | 0.106 | 0.056 | -0.003,0.215 | | 0.046 | 0.073 | -0.097,0.189 | -0.043 | 0.056 | -0.153,0.067 | 0.262** | 0.088 | 0.09,0.434 |
| FW_severe | -0.614*** | 0.075 | -0.761,-0.467 | | -0.974*** | 0.099 | -1.168,-0.78 | -0.977*** | 0.077 | -1.128,-0.825 | -0.171 | 0.11 | -0.387,0.045 |
| FW_moderate | -0.133** | 0.052 | -0.234,-0.032 | | -0.151* | 0.07 | -0.289,-0.014 | -0.209*** | 0.054 | -0.315,-0.103 | 0.085 | 0.082 | -0.075,0.245 |
| Variable*OOP | -0.069*** | 0.017 | -0.101,-0.036 | | 0.038* | 0.015 | 0.009,0.066 | 0.047** | 0.015 | 0.018,0.076 | -0.082*** | 0.021 | -0.123,-0.041 |
| Variable*OS | 0.027*** | 0.007 | 0.013,0.041 | | -0.006 | 0.007 | -0.021,0.009 | 0 | 0.007 | -0.014,0.015 | 0.034*** | 0.01 | 0.015,0.053 |
| Variable*HG | -0.011*** | 0.003 | -0.017,-0.005 | | 0.006 | 0.003 | 0,0.012 | 0.013*** | 0.003 | 0.007,0.019 | -0.022*** | 0.004 | -0.03,-0.013 |
| Variable*Rash | -0.005 | 0.004 | -0.013,0.004 | | 0.005 | 0.005 | -0.004,0.014 | -0.006 | 0.004 | -0.014,0.003 | 0 | 0.006 | -0.012,0.013 |
| Variable*Pain_severe | -0.946*** | 0.114 | -1.169,-0.723 | | 0.349** | 0.125 | 0.103,0.594 | 0.624*** | 0.114 | 0.402,0.847 | -1.038*** | 0.168 | -1.368,-0.708 |
| Variable*Pain_moderate | -0.123 | 0.086 | -0.291,0.045 | | 0.01 | 0.091 | -0.168,0.188 | 0.220** | 0.084 | 0.055,0.385 | -0.374** | 0.128 | -0.625,-0.122 |
| Variable*FW_severe | -0.547*** | 0.112 | -0.767,-0.328 | | 0.225 | 0.12 | -0.011,0.46 | 0.320** | 0.114 | 0.096,0.545 | -1.203*** | 0.159 | -1.515,-0.892 |
| Variable*FW_moderate | 0.015 | 0.081 | -0.144,0.173 | | 0.015 | 0.086 | -0.153,0.183 | 0.172* | 0.079 | 0.017,0.328 | -0.367** | 0.119 | -0.6,-0.133 |
| **SD** | | | | | | | | | | | | | |
| const | 1.788*** | 0.107 | 1.579,1.997 | | 1.410*** | 0.125 | 1.165,1.654 | 1.612*** | 0.117 | 1.383,1.842 | 1.589*** | 0.102 | 1.389,1.789 |
| OS | 0.013* | 0.005 | 0.002,0.024 | | 0.060*** | 0.005 | 0.051,0.07 | 0.061*** | 0.004 | 0.053,0.07 | -0.024*** | 0.007 | -0.037,-0.01 |
| Hyperglycaemia | 0.015*** | 0.003 | 0.01,0.02 | | 0.015*** | 0.003 | 0.009,0.021 | 0.017*** | 0.002 | 0.013,0.022 | -0.014*** | 0.003 | -0.02,-0.008 |
| Rash | 0.002 | 0.005 | -0.008,0.011 | | -0.003 | 0.005 | -0.012,0.006 | -0.004 | 0.004 | -0.012,0.004 | 0.006 | 0.004 | -0.002,0.014 |
| Pain_severe | 0.805*** | 0.063 | 0.683,0.928 | | 1.032*** | 0.068 | 0.9,1.165 | 1.072*** | 0.057 | 0.961,1.184 | 0.869*** | 0.077 | 0.718,1.021 |
| Pain_moderate | -0.156 | 0.085 | -0.323,0.011 | | 0.038 | 0.12 | -0.198,0.273 | 0.014 | 0.105 | -0.191,0.219 | 0.162 | 0.095 | -0.023,0.348 |
| FW_severe | 0.889*** | 0.061 | 0.77,1.009 | | 0.961*** | 0.059 | 0.847,1.076 | 0.963*** | 0.061 | 0.845,1.082 | 0.841*** | 0.058 | 0.727,0.955 |
| FW_moderate | 0.029 | 0.064 | -0.097,0.154 | | -0.049 | 0.059 | -0.165,0.068 | -0.007 | 0.06 | -0.124,0.11 | -0.002 | 0.059 | -0.117,0.114 |
| Variable*OOP | 0.234*** | 0.016 | 0.203,0.264 | | 0.168*** | 0.012 | 0.145,0.192 | 0.165*** | 0.015 | 0.136,0.193 | 0.266*** | 0.017 | 0.233,0.299 |
| Variable*OS | 0.096*** | 0.006 | 0.083,0.108 | | 0.01 | 0.006 | -0.002,0.022 | 0.008 | 0.01 | -0.011,0.028 | 0.085*** | 0.006 | 0.073,0.097 |
| Variable*HG | 0.012** | 0.004 | 0.004,0.02 | | -0.008 | 0.007 | -0.022,0.006 | -0.001 | 0.004 | -0.01,0.007 | 0.016*** | 0.004 | 0.009,0.023 |
| Variable*Rash | -0.004 | 0.005 | -0.014,0.006 | | -0.003 | 0.005 | -0.013,0.007 | -0.008 | 0.008 | -0.024,0.009 | -0.002 | 0.005 | -0.011,0.008 |
| Variable*Pain_severe | 1.095*** | 0.115 | 0.87,1.319 | | 0.576*** | 0.11 | 0.36,0.792 | 0.092 | 0.172 | -0.245,0.428 | 1.074*** | 0.118 | 0.843,1.305 |
| Variable*Pain_moderate | 0.153 | 0.135 | -0.111,0.418 | | 0.273* | 0.109 | 0.06,0.486 | 0.145 | 0.13 | -0.11,0.399 | -0.326** | 0.105 | -0.532,-0.119 |
| Variable*FW_severe | 0.431*** | 0.102 | 0.231,0.632 | | 0.071 | 0.145 | -0.214,0.355 | -0.033 | 0.119 | -0.266,0.2 | 0.424*** | 0.119 | 0.191,0.658 |
| Variable*FW_moderate | -0.068 | 0.085 | -0.235,0.1 | | 0.031 | 0.069 | -0.105,0.166 | 0.036 | 0.094 | -0.148,0.22 | 0.075 | 0.08 | -0.082,0.232 |
| Number of observations | 26928 | | | 26496 | | | | 26928 | | | 26928 | | |
| chi-squared | 2683.848 | | | 2475.613 | | | | 2482.438 | | | 2593.99 | | |
| Model degrees of freedom | 33 | | | 33 | | | | 33 | | | 33 | | |
| Log likelihood | -7087.22 | | | -7074.47 | | | | -7182.67 | | | -7089.46 | | |
| * p<0.05 ** p<0.01 *** p<0.001 | | | | | | | | | | | | | |

Table 5: Mean utility scores per latent class

| Class | Number of patients | Mean utility (Std.dev) |
| --- | --- | --- |
| Money indifferent | 216 | 0.73(0.28) |
| Pay anything for longer life | 89 | 0.60(0.37) |
| Rational decision maker | 203 | 0.56(0.33) |
| Treatment avoiders | 53 | 0.58(0.38) |
